# Body Image and Emotional Status in Patients with Acquired Brain Injury

**DOI:** 10.3390/jcm12124070

**Published:** 2023-06-15

**Authors:** Viviana Lo Buono, Francesco Corallo, Lilla Bonanno, Angelo Quartarone, Maria Cristina De Cola

**Affiliations:** IRCCS Centro Neurolesi Bonino-Pulejo, 98124 Messina, Italy; francesco.corallo@irccsme.it (F.C.); lilla.bonanno@irccsme.it (L.B.); mariacristina.decola@irccsme.it (M.C.D.C.)

**Keywords:** acquired brain injury, body image, self-perception, depression, anxiety

## Abstract

Emotional experiences can lead to a real or distorted self-representation. After brain damage, altered self-perception of one’s own body image is frequent. This study evaluates the relationship of mood disorders and lesion sites on body image in a cohort of ABI patients. A total of 46 patients (26 men, 20 women) without severe physical impairments were found eligible for this study. Patients underwent Beck’s Depression Inventory and the Hamilton Rating Scale for Anxiety to assess mood disorders, whereas the Body Image Scale and Human Figure Drawing were used to evaluate body dissatisfaction and implicit body image. The Montreal Cognitive Assessment was used to assess patients’ cognitive condition. We found a moderate correlation between depression and body image (r = 0.48), as well as between anxiety and body image (r = 0.52), and the regression model also reported the right lesion site as a predictive variable for body image score. In addition, the regression model built by Human Figure Drawing scores showed anxiety, cognitive functioning, and a marital status of single to be significant predictors. The study confirmed that participants with acquired brain injury have deficits in body representation associated with mood disorders, regardless of the side of the lesions. A neuropsychological intervention could be useful for these patients to improve their cognitive performance and learn to manage emotional dysfunction in order to increase their self-perception of body image and improve their quality of life.

## 1. Introduction

Acquired brain injury (ABI) defines numerous conditions that affect clinical outcomes and quality of life in survivors. The most frequent causes of ABI are traumatic brain injury, stroke, and hypoxic–ischemic encephalopathy after cardiac arrest [1].

Cerebral damage can be focal or diffuse and can present with different variations in severity and location within the brain, leading to a multitude of possible changes in functioning. The emotional and psychiatric consequences of ABI can have an important impact on individual and familial well-being [2]. Psychological alterations, such as distress, depression, anxiety, and post-traumatic stress disorder, are frequently diagnosed in ABI patients [3]. Moreover, these patients can present with impairments in self-monitoring and control such as the inability to differentiate emotions, revealing various symptoms of emotional dysregulation including disinhibited emotion/behavior and reduced emotional awareness and expression [4]. Such mood alterations may depend on the brain injury location, pre-existing mental health condition, personality type, psychological stressors, and family and social support. The changes in mood, emotions, and motivation are also related to alterations in the flow of excitatory and inhibitory neurotransmitters in the brain networks involved in modulating emotions and behavior [5]. In addition, the onset of depressive and anxious symptoms may represent a response to the damage, grief, and regret of the previous life condition. ABI can cause emotional and cognitive impairment, but physical disability is also a frequent sequela. Motor deficits, hemiplegia, balance, and coordination impairments are often described in ABI patients [6].

ABI patients face many losses (e.g., physical, cognitive, and social) that can impact self-image. One of the many adjustments that people with ABI have to make is coping with the changes in body functioning and appearance [7]. Body representation includes body schema and body image. Body schema (the unconscious representation of the body with perceptive functions) is the dynamic representation of body parts and their relative positions in the space, and it contributes to the execution of movements by providing information about the dynamic position of each body part in relation to other parts [8]. Body image is “the picture of our own body which we form in our mind, that is to say, the way in which the body appears to ourselves”, to use Schilder’s words [9]. 

Teal and Athelstan [10] defined body image the psychological experience focusing on feelings and attitudes toward one’s own body. Body image includes both conscious and unconscious feelings, and these may relate to the size, function, and appearance of one’s body [11,12]. According to Norris [13], body image is also seen as a social construct that is inextricably linked to personality, ego, identity, and feeling of value. Many people accept only a “normal” appearance, according to society’s definition of normalcy. Therefore, body image is a complex psychological dimension that develops through the bodily experience of the individual. It represents a construct enclosing body-related perceptions and self-attitudes, such as thoughts, beliefs, feelings, and behaviors. A key feature of our body representation is that, even after development in early childhood, it is flexible enough to adapt to changes in size and experience throughout our lives [14]. 

This representation of the body is implicated in numerous psychological, neurological, and physical conditions. It is well known that the neuroanatomical basis influencing body representations includes an integrated system of cerebral areas and functional networks. The main brain regions involved in body representation are the thalamus, insula, and cerebellum [15]. On the other hand, the perception and ongoing knowledge of one’s body comes from different receptors and sensory modalities. This perceptual integration process is generally associated with the cerebral networks of the right inferior parietal lobule, left inferior temporal sulcus, and left middle frontal gyrus [16].

Alterations in motor and sensory cortical processing generate inaccurate body information, which can affect the perception of limb size, position, shape, or weight. Some studies have reported that patients with arm paresis showed transient overestimation of the features of the contralateral paretic arm after stroke, and described it as longer or larger than it actually was [17].

Physical pain or pain syndrome, psychological distress [18], mood disorders, relational competence, and social and occupational functions affect body image since they can cause disturbances in several components of self-concept and the loss of self-identity [19]. Affectivity could also play an important role in body image. Cooper and Taylor [20] found that a negative mood state of mind leads to greater disturbances in body perception. However, not only neurological patients but also healthy individuals can have a distorted view of body image. Linkenauger et al. [21] showed that right-handed participants judged their right arm to be longer than their left arm, even though there was actually no difference in length between the two. Therefore, the specific psychopathology of body image can span from disorders in body perception to alterations due to its cognitive–affective dimensions. 

Although the scientific literature includes several works reporting an association between body image disturbance and some mental health outcomes, such as depression and psychiatric disorders [22,23], there are very few studies conducted on adult patients with an ABI [16,24,25,26].

In this study, we analyzed the factors related to body image; in particular, we explored the relationship between body image perception and emotional disorders in ABI patients, focusing on lesion sites and some demographic characteristics that might affect emotional behaviors.

## 2. Materials and Methods

### 2.1. Study Population

This was a cross-sectional study. The population study included 46 ABI patients (26 men, 20 women) hospitalized at the Rehabilitation Unit for Severe Acquired Brain Injuries of the IRCCS Centro Neurolesi “Bonino-Pulejo” of Messina from January 2019 to December 2019. The study protocol was approved by the Local Ethics Committee according to Declaration of Helsinki. All participants signed written informed consent upon hospital admission to use their information for research purposes. All information related to ABI was collected by means of interviews, examination of medical records, and psychometric tests. Demographic and clinical data are presented in Table 1.

Subjects were selected according to the following inclusion criteria: (i) ABI diagnosis within 1–3 months after onset from the brain injury occurrence; (ii) age over 17 years; and (iii) no previous history of neurological or psychiatric disorder. We excluded patients with: (i) prosopagnosia or neglect; (ii) paresis, paraplegia, or tetraplegia; or (iii) severe motor disorders and physical impairments of the limbs (Barthel Index score < 75). 

Subjects were divided into three groups according to the lesion site: right lesion, left lesion, bilateral lesion. 

### 2.2. Assessment

At admission, all patients underwent a neuropsychological and mood testing assessment. A battery of tests was administered to each patient by two trained neuropsychologists. We used the Montreal Cognitive Assessment (MoCA), the Beck Depression Inventory II (BDI-II), and the Hamilton Rating Scale for Anxiety (HAM-A). The MoCA is a cognitive screening test for the detection of mild cognitive impairment; a score <26 indicates the presence of cognitive alterations [27]. The BDI-II is a 13-item self-report questionnaire designed to measure the severity of depression symptomatology: a total score of 10–19 indicates mild depression; a score of 20–29 is moderate depression; and a score >30 indicates a severe depressive state [28]. The HAM-A is a 14-item scale measuring the severity of psychic and somatic anxiety. The score ranges from 0 (not present) to 4 (severe) with a total score range of 0–56; a score <17 indicates mild anxiety, a score of 18–24 is moderate anxiety, and a score of 25–30 is severe anxiety [29]. In addition, the neuropsychologists administered the Body Image Scale (BIS) and the Human Figure Drawing (HFD). The BIS scale is a 10-item questionnaire that explores different dimensions of body image. Each question is rated on a 5-point Likert scale (0 = never; 1 = a little; 2 = quite a bit; 3 = very much; 4 = don’t know). The final score is the sum of the 10 items, ranging from 0 to 30; a score of 0 indicates the absence of symptoms or distress, and higher scores correspond to higher distress or more body image concerns [30]. The HFD is used to evaluate various psychological states, especially assessing the psychic status including psychiatric illness and personality state [31]. All patients drew a human figure. After the first drawing, they drew another human figure that is of the opposite sex. In total, 43 particulars related to the human figure (physical characteristics, drawing line, etc.) were evaluated with a 0–1 value, i.e., 1 = presence of the detail, 0 = absence of the detail. All these values contribute to obtaining a total score of the drawings. High scores correspond to satisfactory body representation.

### 2.3. Statistical Analysis

Since both the Shapiro–Wilk test and the graphical inspection showed a not-normal distribution for most of the target variables, nonparametric analysis was performed. Therefore, continuous variables were expressed as median and first-third quartile, whereas categorical variables as were expressed frequencies and percentages. The χ^2^ test with continuity correction or the Fisher’s exact test was used to assess for statistical differences in proportions, when appropriate, whereas the Mann–Whitney U and the Kruskall–Wallis tests were used to compare continuous variables, respectively, comparing pairs and multiple groups. Correlations between variables were calculated using Spearman’s coefficient. Finally, we used the multiple regression analysis to estimate the effects of mood, lesion side, and cognition (independent variables) on body image/implicit body representation (dependent variable) after adjustment for age, sex, and marital status. The backward elimination stepwise procedure for the choice of the best predictive variables according to the Akaike information criterion (AIC) was applied. Regression models were performed both on the whole sample and on lesion side subgroups. Analyses were performed using an open-source R4.0.5 software package, considering a *p* < 0.05 as statistically significant.

## 3. Results

Patients were aged 55.3 ± 17.9 years on average and 56.5% were of the male gender. The injuries were mainly vascular (63%) and unilateral (80.4%), especially on the right side of the brain (47.8%). All patients were right handed. A sociodemographic and clinical description of the patients is reported in Table 1 and Table 2. 

The multiple regression analysis showed lesions on the right side as a significant predictor for a high BIS score, together with HAM-A and BDI-II (Table 3). In addition, multiple regression results reported that HAM-A, MoCA, and a marital status of Single as the only significant predictors for a high HFD score (Table 4).

HAM-A and BDI-II scores resulted to be moderately correlated with BIS: r = 0.48 and r = 0.52, respectively. We did not find a correlation between body image and implicit body representation (r = 0.01), even the net of the other variables (r = −0.01).

### Subgroup Analysis by Lesion Site

As shown in Table 3 and Table 4, we found that BDI-II was a significant predictor for BIS and MoCA for HFD in the right lesion side subgroup, whereas HAM-A was a significant predictor for both BIS and HFD in the left lesion side subgroup. BDI-II was a significant predictor for HFD in bilateral lesion patients, and MoCA for both BIS and HFD.

## 4. Discussion

Body image implies a visual representation of the body through our graphic depiction of ourselves. This representation results from the integration of multisensory neural inputs [32]. In the clinical setting, body image disorders are relatively common and involve both psychiatric and neurological disorders. We aimed to describe the relationship between body perception and emotional disorders within a sample of brain-damaged patients. In ABI patients, body representations may be subject to complex distortions. Moreover, ABI patients may show important alterations in cognitive, sensory, motor, and psychosocial domains, and rates of anxiety and depression are substantially higher than in the general population [33].

In this study, the lesion side seems to be a predictor of body image, together with anxiety and depression, whereas cognitive functioning, anxiety, and marital or single status seem to be predictors of implicit body representation.

The subgroup of ABI patients with right-sided lesions was the most depressed and had the least alteration in implicit body representation, while patients with bilateral lesions were the least anxious and had the highest body image values (although these differences did not reach statistical significance). Notably, depression was a significant predictor for body image in the right lesion patients, and for implicit body representation in bilateral lesion patients, together with cognitive functioning, whereas anxiety was a significant predictor for both body image and implicit body representation in left lesion patients.

The strong relationships we have described between emotional disorders, such as anxiety and depression, and body image reinforce the idea that body dissatisfaction is an important psychological variable affecting mood, both positively and negatively [34]. The body image perception, in the absence of severe cognitive and physical impairment [35], has a significant impact on the quality of life of the patients [36]. A wide range of persistent emotional disorders may occur after ABI, many of which are significantly related to body image perception [37].

Mood alterations may also affect patients’ self-esteem, as hypothesized by Howes et al. [25,26], who found that both males and females with ABI had reduced self-esteem compared with controls: men had lower body dissatisfaction (especially in sexual functioning), and women had higher levels of depression. Therefore, the severe and delicate condition of these patients requires, beyond motor and functional rehabilitation, cognitive and psychological interventions both during and after hospitalization. Maresca et al. [27], in their RCT, proposed a combined approach including psychological counseling and nutritional treatment for ABI inpatients, showing positive changes in body representation and improved mood.

The relationship between lesion site and emotional disorders has been the object of numerous studies [38,39]. Lesions in the left frontal cortex are frequently linked to post-stroke depression and the co-occurrence of anxiety and depression, whereas lesions in the right hemisphere are more frequently connected with isolated anxiety symptoms [40]. However, some authors reported contrasting results and described major depression following right-hemisphere lesions [41]. Regarding how hemisphere lateralization affects emotions and mood regulation, there are two basic theories [42]. In the first, it is assumed that regardless of the emotion’s valence, the right cerebral hemisphere is dominant in its production. According to the second theory, the left hemisphere is specifically designed to produce happy emotions and moods, while the right hemisphere is in charge of moderating negative emotions and mood [43]. 

Patients with right frontal lesions often show inappropriate indifference to negative stimuli or inappropriate euphoria that may be related to the presence of anosognosia, a lack of awareness of physical or cognitive disability following brain damage [44]. Instead, depression is most frequently found in patients with lesions in the right parietal lobe [45].

Lesions in the right hemisphere have also been associated with spatial deficit and changes in body representations [46]. However, neuroimaging studies on cerebral dysfunctions have described neurophysiologic abnormalities in multiple areas of the orbital and medial prefrontal cortex, including amygdala and related parts of the striatum and thalamus [47]. It would seem that the ‘deactivation’ of these areas in depressed mood reflects neurophysiological interactions between cognitive and emotional processing. Therefore, emotional disorders and alterations in body image could be related to damage in brain networks rather than individual cerebral areas. 

We also found a strong relation between implicit body representation and cognitive functioning: the more cognitive deterioration increases, the more implicit body representation decreases and emotional disorders begin to increase, especially in singles. This relationship deserves to be investigated more deeply, as emotional aspects affect relational ones, especially in terms of the support that caregivers can provide. However, limited research exists regarding the effects of body dissatisfaction within the marital dyad.

Body representation is one of the components of personal identity. Previous research has described that gradual changes in a disease process in disability may allow gradual adaptation to changes in body image and may be less psychologically traumatic than a sudden insult to the body [48].

In ABI patients, the adaptation to changes in body image appears to be different since experiencing a sudden onset of alterations. Body image change in ABI patients requires a process of adapting to an altered identity, a new identity as “sick”, resulting in the belief that they have no control over their health. 

Alterations in body image more broadly reflect a condition of physical and mental distress that requires a necessary, and sometimes complicated, process of adaptation to the new living condition. Body image in people with physical disability is an important area of investigation which has received little attention in the literature. Clinicians should pay more attention to the psychological implications of ABI, as they are known to impair recovery and interfere with rehabilitation. Currently, the main focus of rehabilitation is on improving physical function and restoring, as much as possible, the previous level of functioning. Problems of body image and self-esteem can have a pervasive impact on a person’s well-being and should not be overlooked in determining good rehabilitation training. A multidisciplinary approach, including psychological interventions, could aim to identify the person’s premorbid coping style and psychological resources in an attempt to understand and improve their current ability to cope with the disability. Coping strategies refer to the specific efforts, both behavioral and cognitive, that people use to master, tolerate, reduce, or minimize stressful events. Coping is a dynamic process constituted by a series of reciprocal responses through which the individual and environment influence each other reciprocally. Depending on the success or failure of this process, coping may be defined as functional (adaptation) or dysfunctional (increased stress). In functional coping skills, it has been observed that promoting active, problem-focused coping strategies improves psychological outcomes in people with chronic diseases [49,50]. The ability to conceptualize problems related to body image is also a useful starting point for enabling patients to cope with altered body image.

This study presents many limitations, such as the small size of the sample and the study design used to collect data. Therefore, future research should include a larger cohort of patients, e.g., followed in a prospective study with pre–post hospitalization assessments and compared to a control group.

## 5. Conclusions

Cognitive processes and social experiences contribute to the development of body image, which is influenced not only by actual data about one’s physical condition or psychological, emotional, and personal perceptions, but rather by the close interaction between these components and other social factors.

The literature reports that the right hemisphere is involved in body image perception and depressive disorders. However, it is currently difficult to determine whether changes in body image representation and mood status are due to the same neurological substrate, or whether emotional disorders are a response to an altered body image or vice versa. In this study, we confirmed the important relationship between altered mood and body image after brain damage, regardless of the side of the lesions. Indeed, the change in body image seems to be the consequence of unilateral brain damage on both the right and left sides, affecting interoceptive perceptions and their link to emotion regulation.

## Figures and Tables

**Table 1:** Sociodemographic description of the sample.

Characteristics	All (n = 46)	Lesion Side			
		Right (n = 22)	Left (n = 15)	Bilateral (n = 9)	*p*-Value
Age (years)	55.5 (46.2–67.7)	52.0 (43.5–64.7)	56.0 (49.5–65.0)	64.0 (50.0–78.0)	0.348
Male	26 (56.5)	13 (59.1)	6 (40.0)	7 (77.8)	0.184
Education					0.354
None	1 (2.2)	0 (0.0)	0 (0.0)	1 (11.1)	
Elementary school	4 (8.7)	3 (13.6)	1 (6.7)	0 (0.0)	
Middle school	21 (45.6)	10 (45.5)	6 (40.0)	5 (55.6)	
High school	15 (32.6)	8 (36.4)	5 (33.3)	2 (22.2)	
Vocational school	1 (2.2)	1 (4.5)	0 (0.0)	0 (0.0)	
University degree	4 (8.7)	0 (0.0)	3 (20.0)	1 (11.1)	
Marital status					0.987
Single	13 (28.3)	6 (27.3)	5 (33.3)	2 (22.2)	
Married	25 (54.3)	12 (54.5)	7 (46.7)	6 (66.7)	
Divorced/Separated	3 (6.5)	2 (9.1)	1 (6.7)	0 (0.0)	
Widowed	5 (10.9)	2 (9.1)	2 (13.3)	1 (11.1)	

Continuous variables are expressed as median (first-third quartile), whereas categorical variables are expresesd as frequencies (percentages).

**Table 2:** Clinical description of the sample.

Characteristics	All (n = 46)	Lesion Side			*p*-Value
		Right (n = 22)	Left (n = 15)	Bilateral (n = 9)	
Etiology					0.721
Vascular	29 (63.0)	15 (68.2)	8 (53.3)	6 (66.7)	
Traumatic	17 (37.0)	7 (31.8)	7 (46.7)	3 (33.3)	
MoCA score	19.0 (14.2–22.0)	18.0 (15.0–21.7)	21.0 (14.5–22.0)	19.0 (13.0–22.0)	0.923
HAM-A score	11.5 (6.2–15.7)	12.0 (7.2–18.2)	11.0 (6.5–15.0)	9.0 (6.2–15.7)	0.616
BDI-II score	16.0 (8.5–18.0)	16.5 (11.7–23.7)	11.0 (6.0–17.5)	13.0 (10.0–17.0)	0.126
BIS score	7.0 (2.0–13.0)	6.0 (1.2–15.0)	6.0 (1.5–12.5)	9.0 (7.0–13.2)	0.571
HFD score	33.0 (24.2–45.7)	30.5 (21.0–44.5)	34.0 (29.5–45.5)	34.0 (17.0–46.0)	0.721

Continuous variables are expressed as median (first-third quartile), whereas categorical variables are expressed as frequencies (percentages).

**Table 3:** Backward linear regression: predictors for body image.

Whole Sample	Estimation	Std Error	*p*-Value	Adjusted R^2^
Lesion side—Left	−1.037	2.604	0.693	0.496
Lesion side—Right	−5.144	2.469	**0.044**	
HAM-A	0.413	0.136	**0.004**	
BDI-II	0.448	0.139	**0.002**	
Left lesion side subgroup				
Age	−0.117	0.085	0.192	0.717
HAM-A	1.033	0.171	**<0.001**	
Right lesion side subgroup				
BDI-II	0.697	0.146	**<0.001**	0.509
Bilateral lesion subgroup				
Age	−0.246	0.133	0.139	0.734
BDI-II	0.302	0.213	0.228	
MoCA	−1.333	0.427	**0.035**	

Legend: HAM-A = Hamilton Rating Scale for Anxiety; BDI-II = Beck Depression Inventory II. Statistical significances are in bold.

**Table 4:** Backward linear regression: predictors for implicit body representation.

Whole Sample	Estimation	Std Error	*p*-Value	Adjusted R^2^
Age	−0.259	0.143	0.077	0.411
HAM-A	0.606	0.289	**0.043**	
Marital status—Single	−20.845	8.483	**0.019**	
BDI-II	−0.502	0.263	0.064	
MoCA	1.802	0.343	**<0.001**	
Left lesion side subgroup				
Age	−0.579	0.172	**0.006**	0.451
HAM-A	1.160	0.436	**0.022**	
BDI-II	−0.637	0.489	0.219	
Right lesion side subgroup				
HAM-A	0.481	0.353	0.189	0.316
MoCA	2.039	0.615	**0.004**	
Bilateral lesion subgroup				
HAM-A	0.494	0.319	0.182	0.942
BDI-II	−0.869	0.307	**0.037**	
MoCA	2.966	0.298	**<0.001**	

Legend: HAM-A = Hamilton Rating Scale for Anxiety; BDI-II = Beck Depression Inventory II; MoCA = Montreal Cognitive Assessment. Statistical significances are in bold.

## Data Availability

The data that support the findings of this study are available on request from the corresponding author.
